# EpCAM tumor specificity and proteoform patterns in urothelial cancer

**DOI:** 10.1007/s00432-023-04809-9

**Published:** 2023-05-08

**Authors:** Franz F. Dressler, Sofie Hinrichs, Marie C. Roesch, Sven Perner

**Affiliations:** 1 Institute of Pathology, Charité-Universitätsmedizin Berlin, Corporate Member of Freie Universität Berlin, Humboldt-Universität zu Berlin and Berlin Institute of Health, Charitéplatz 1, 10117 Berlin, Germany; 2 Institute of Pathology, University Medical Center Schleswig-Holstein, Campus Lübeck, Lübeck, Germany; 3 Department of Urology, University Hospital Schleswig-Holstein, Campus Lübeck, Lübeck, Germany; 4 Institute of Pathology, Research Center Borstel, Leibniz Lung Center, Borstel, Germany; 5 Institute of Pathology and Hematopathology, Hamburg, Germany

**Keywords:** EpCAM, EpCAM fragments, Urothelial cancer, Bladder cancer, Biomarker, Tumor specificity, Oncology, Protein quantitation, Targeted therapeutics, Precision medicine, Translational oncology

## Abstract

**Background:**

The role of the epithelial cell adhesion molecule (EpCAM) in cancer is still unclear. EpCAM cleavage through regulated intramembrane proteolysis results in fragments which interact with both oncogenic and tumor suppressive pathways. Additionally, the EpCAM molecule itself is used as a descriptive therapeutic target in urothelial cancer (UC), while data on its actual tumor specificity remain limited.

**Methods:**

Samples from diagnostic formalin-fixed paraffin-embedded (FFPE) UC tissue and fresh-frozen UC cells were immunoblotted and used for qualitative characterization of five different EpCAM fragments. These expression patterns were quantified across a cohort of 76 samples with 52 UC and 24 normal urothelial samples. Cell viability effects of the extracellular EpEX fragment were assessed in the UC cell lines T24 and HT1376.

**Results:**

The proteolytic EpCAM fragments could be identified in clinical FFPE tissue specimens too. Neither overall nor fragment-specific EpCAM expression showed relevant tumor specificity. EpEX and its deglycosylated variant showed an inverse relationship across healthy and tumor tissue with a decrease of deglycosylated EpEX in tumors. However, extracellular EpEX did not show a relevant effect in vitro.

**Conclusions:**

EpCAM should not be regarded as tumor-specific in UC without patient-specific predictive testing. EpCAM fragment patterns indicate cancer-specific changes and could be involved in its complex tumor-biological role.

**Supplementary Information:**

The online version contains supplementary material available at 10.1007/s00432-023-04809-9.

## Introduction

Since the discovery of the epithelial cell adhesion molecule (EpCAM) as a tumor marker in 1979 (Herlyn et al. 1979), many studies have investigated its structure and function. Physiologically, EpCAM influences cortical tension in cells, thereby maintaining the epithelial barrier (Lei et al. 2012; Schnell et al. 2013a, b). EpCAM has also been used as epithelial marker to detect circulating tumor and cancer stem cells (Alix-Panabieres and Pantel 2013; Joosse et al. 2015).

EpCAM has been attributed a multi-faceted role in tumors. Cleavage via regulated intramembrane proteolysis results in the production of two main EpCAM fragments: the extracellular (EpEX) and the intracellular fragment (EpIcD) (Maetzel et al. 2009; Lei et al. 2012; Schnell et al. 2013a, b). These fragments as well as full-length EpCAM interact with different cell signaling pathways (Gires et al. 2020, Mohtar Syafruddin et al. 2020). EpEX has been shown to promote proliferation through binding at the epithelial growth factor receptor (EGFR), Akt phosphorylation and induction of EKR1/2, and to regulate epithelial-to-mesenchymal transition (Kuiper et al. 2011; Sankpal et al. 2017; Pan et al. 2018). EpIcD forms a complex with beta-catenin and FHL2, relocates to the nucleus, and induces transcription of *MYC* and other oncogenes (Maetzel et al. 2009; Schnell et al. 2013a, b, Mohtar et al. 2020). Furthermore, deletions at the 3’-end of *EPCAM* lead to transcriptional silencing of *MSH2* and result in Lynch syndrome (Niessen et al. 2009; Kuiper et al. 2011; Ligtenberg et al. 2013).

Evaluation of EpCAM as a prognostic marker led to mixed results. Its expression correlated inversely with disease- and progression-free survival in breast cancer and with overall survival in colorectal and gall bladder cancer (Spizzo et al. 2004; Varga et al. 2004; Seeber et al. 2016; Gires et al. 2020). In renal and thyroid carcinoma (Seligson et al. 2004; Went et al. 2005; Ensinger et al. 2006), however, increased EpCAM expression correlated with better prognosis. EpCAM has also been used as a descriptive target for antibody-dependent cellular cytotoxicity (ADCC) in breast and prostate cancer with some (weak) antitumor activity (Gottlinger et al. 1986; Schmidt et al. 2010, 2012; Eyvazi et al. 2018; Keller et al. 2019).

In urothelial carcinoma (UC), increased expression of EpCAM has also been reported (Brunner et al. 2008). Urinary EpCAM has been linked to advanced stages and reduced survival (Bryan et al. 2014). Compared to other malignancies, a high rate of nuclear relocation of EpIcD has been suggested for UC (Fong et al. 2014). Using EpCAM as a descriptive target, a cytotoxic antibody–drug conjugate (ADC) has been developed for intravesical instillation and phase II clinical trials were conducted (Kowalski et al. 2010, 2012). However, data on the tumor specificity of EpCAM in UC and cancer in general remain sparse and are based on immunohistochemistry (Momburg et al. 1987; Zorzos et al. 1993, 1995; Schmelzer 2008).

With its molecular structure deciphered (Pavsic et al. 2014), proteolytic EpCAM fragments have been described in cell lines (Schnell et al. 2013a, b; Schnell et al. 2013a, b). These results have hitherto not been matched to tissue, including formalin-fixed paraffin-embedded (FFPE) specimens as used in routine diagnostics. Closing this gap, this study aims to evaluate the distribution of EpCAM and its fragments qualitatively and quantitatively in a clinical cohort of UC samples. Also, the actual tumor specificity of EpCAM is assessed by comparison to corresponding healthy tissue.

## Materials and methods

### Tissue macrodissection

The FFPE tissue blocks were cut into serial 20 µm sections and placed on slides. A separate section was stained with hematoxylin and eosin (HE). The stained slides were evaluated by a pathologist and areas of tumor and healthy mucosa were marked. The number of sections used for protein extraction were chosen to yield a cumulative area of 1 cm^2^. The macrodissected tissue was transferred to 2 ml low bind tubes (88,379 ThermoFisher, Waltham USA) and stored at 4 °C in the dark.

### Protein extraction and quantification

Samples were prepared as previously described (Dressler et al. 2022). Briefly, samples were deparaffinized twice with 2 ml xylol for 15 min followed by centrifugation at 2 000 G for 2 min and xylol removal. Samples were then washed with double-distilled water for 30 s and centrifuged again. After transfer to 0.2 ml PCR tubes, 60 µl extraction buffer was added [protease inhibitor 0.94% v/v (78,440 Thermo Scientific, Waltham USA), beta-mercaptoethanol 4.7% v/v, sodium dodecyl sulfate 2% w/v, Tris-base 200 mM, EDTA 1 mM, pH 7.20]. Samples were incubated on ice for 5 min, vortexed, placed in a thermocycler, and then incubated at 4 °C for 5 min, 90° C for 90 min and four cycles of 99 °C for 5 min followed by 60 °C for 10 min. The samples were vortexed again and undissolved parts were removed by centrifugation at 10 000 G for 15 min. The supernatant extracts were stored at – 80 °C.

For the protein concentration measurements, the EZQ quantification kit (R33200, Invitrogen ThermoFisher, Waltham USA) was used as per the manufacturer’s instructions. Membranes were imaged on a Gel Doc XR + imaging system (1,708,195, BioRad, Feldkirchen, Germany). Images were quantified in ImageLab (version 6.0.1, BioRad). Data processing and linear regression were performed in Excel (version 16.64 OS; Microsoft, Seattle, USA).

### Immunoblotting

Immunoblotting was performed using 12% SDS MOPS Bis–Tris gels (NW00105BOX, ThermoFisher Scientific, Waltham USA) with the Xcell SureLock™ system (EI0001 ThermoFisher Scientific, Waltham USA) at 200 V (maximum 250 mA) for 47 min. Based on the linear dynamic range of the blot signal (Supplemental Fig. S1), depending on extract protein concentration, at least 10 µg were loaded per lane (maximum 30 µg). Samples were transferred at 30 V for 60 min to a nitrocellulose membrane with 0.45 µm pore size (LC2001 ThermoFisher Scientific, Waltham USA). The membrane was then washed with distilled water and blocked in 5% w/v milk in phosphate buffer saline containing 0.5% v/v Tween-20 (PBST-M) or 5.5% w/v bovine serum albumin (BSA) in phosphate buffer saline containing 0.5% v/v Tween-20 (PBST-BSA). Membranes were cut and the washed twice with BSA- and milk-free PBST. Primary incubation was done overnight using the mouse IgG anti-EpCAM monoclonal antibody MOC31 (binding to amino acids (AA) 27–56 in the N-terminal domain of EpCAM; 1:150 NBP2-44,640, Novus Biologicals, Littleton, USA), rabbit IgG anti-EpCAM monoclonal antibody E144 (binding to AAs 266–314; 1:1000, ab32392 abcam, Cambridge UK), with the rabbit IgG anti-integrin beta monoclonal antibody (1:1000 #4706, Cell Signaling Technology, Danvers, USA) and rabbit IgG anti-cofilin-1 monoclonal antibody (1:1000 #5175S, Cell Signaling Technology, Danvers USA) as loading controls. Following primary incubation, the membranes were washed with PBST four times and incubated with the secondary antibodies anti rabbit IgG (1:2500, #31,460, ThermoFisher Scientific, Waltham, USA) and anti-mouse IgG (1:2500, #31,430, ibid). Membranes were then washed with three times and pure PBS once, each time for 4 min. The membranes were then reassembled and developed on a densitometric imager (Amersham Imager 600, GE Healthcare 29,083,461, Freiburg, Germany) using enhanced chemiluminescence developing agents (32,106 ThermoFisher Scientific, Waltham, USA). To investigate the specificity of the observed bands samples were also incubated with the anti-EpCAM primary antibody C10 (1:100, sc-25308, Santa Cruz Biotechnology, Dallas, USA) and anti-HPRT (1:100, sc-376938, Santa Cruz Biotechnology, Dallas, USA).

VU1D9 (1:100, NBP2-33,051–0.02 mg, Novus Biologicals, Littleton USA; similar to MOC31) and the polyclonal antibody P6052 (1:1000–1:10,000; similar to E144; kindly provided by B. Giepmans and colleagues, University Medical Center Groningen, Groningen, The Netherlands) exhibited either lower specificity or sensitivity in FFPE tissue of our cohort.

### Immunohistochemistry

Four representative muscle-invasive UC specimens were subjected to immunohistochemistry (IHC) as previously described (Braun et al. 2012). The OptiView Diaminobenzidine (DAB) detection kit was run on a Ventana Benchmark Ultra automated IHC system (Roche, Basel, Switzerland). The slides were evaluated visually by two investigators without disclosure of the IHC antibody. As an IHC alternative for E144, the anti-EpCAM antibody 4A7 (1:100; ab224826 abcam, Cambridge, UK) was used as well as MOC31 (1:100, 790–4561, Ventana, Tuscon, USA).

### Specimen collection

The study was conducted in accordance with the Declaration of Helsinki and with approval of the local ethics committee of the University of Luebeck (19–321). Samples were selected based on the availability of pairs of healthy and tumor tissue.

### Cell culture and proliferation assay

HT1376 and T24 cells were freshly purchased from Merck (87,032,402-1VL, Darmstadt, Germany) and Cell Lines Services (300,352; Eppelheim, Germany). Cells were cultured in DMEM medium (Life Technologies 11,320,033, Carlsbad, USA) with fetal bovine serum 5% (v/v; Biowest S1810-500, Nuaillé, France), L-Glutamine 2 mM (Merck 59202C), and penicillin/streptomycin 1% (v/v; Life Technologies 15,140,122). Cells were detached with Accutase (Thomas Geyer 8,000,921, Berlin, Germany). The MTT assay was performed as kit and per the manufacturer’s instructions (ATCC 30-1010 K, Manassas, USA) with a seeding concentration of 4,500 per well in 90 μl quintuplicates. After incubation for 24 h, the medium was changed and supplemented with EpEX (Abcam 269,992; Cambridge, UK) with adjusted levels of the EpEX buffer [NaCl 0.64% (w/v); KCl 0.02% (w/v); glycerol 20% (v/v); NaH_2_PO_4_ 0.13% (w/v); pH = 7.40]. Due to different proliferation rates, T24 cells were grown for 0, 24, 48, and 72 h, whereas HT1376 cells were grown for 0, 24, 72, and 144 h.

### Proteomic validation cohort

Overlapping with the cohort from this study, pre-existing liquid chromatography-coupled tandem mass spectrometry data (LC–MS/MS; (Dressler and Végvári 2022)) was evaluated for overall EpCAM expression.

### Statistical analysis

All statistical analysis and data visualization were performed in Python (2.7.17 and 3.8.8) using the packages NumPy 1.16.1, SciPy 1.2.2, matplotlib 2.2.4, seaborn 0.9.1 and pandas (0.24.2). Data are generally reported as median with inter-quartile range. Statistical significance was calculated using the Mann–Whitney U test as implemented in the scipy.stats module. Survival analysis was performed using Kaplan–Meier models as implemented in the lifelines module (version 0.26.0) (Davidson-Pilon 2019) with Wilcoxon-weighted log-rank tests for significance.

### Immunoblot quantification

Lane profiles and band intensities were extracted with ImageLab (version 6.1, BioRad, Hercules, USA) with manual lane and band selection. Visual artifacts such as air bubbles, membrane damage, or aberrant electrophoresis, led to exclusion of the respective lanes or bands.

Each 15-lane gel included up to three pooled standards. For each protein and blot, standards were first normalized to the loading control, and their median ratio used to adjust the respective sample intensities (to account for different densitometric intensities across different runs). The individual sample bands were then normalized to their loading control. Cross-gel comparisons were made only with these normalized values. Of the two size controls, cofilin-1 was chosen for normalization due to its invariant expression across samples and tumor stages [data from (Dressler and Végvári 2022)]. For the qualitative evaluation of the superimposed lane profiles, we used both control peaks to align the profiles (Fig. 2A). After baseline subtraction, the profiles were then normed to the area under the curve, enabling relative comparison of the peak pattern.

## Results

### EpCAM variants in diagnostic tissue

**Fig. 1:**
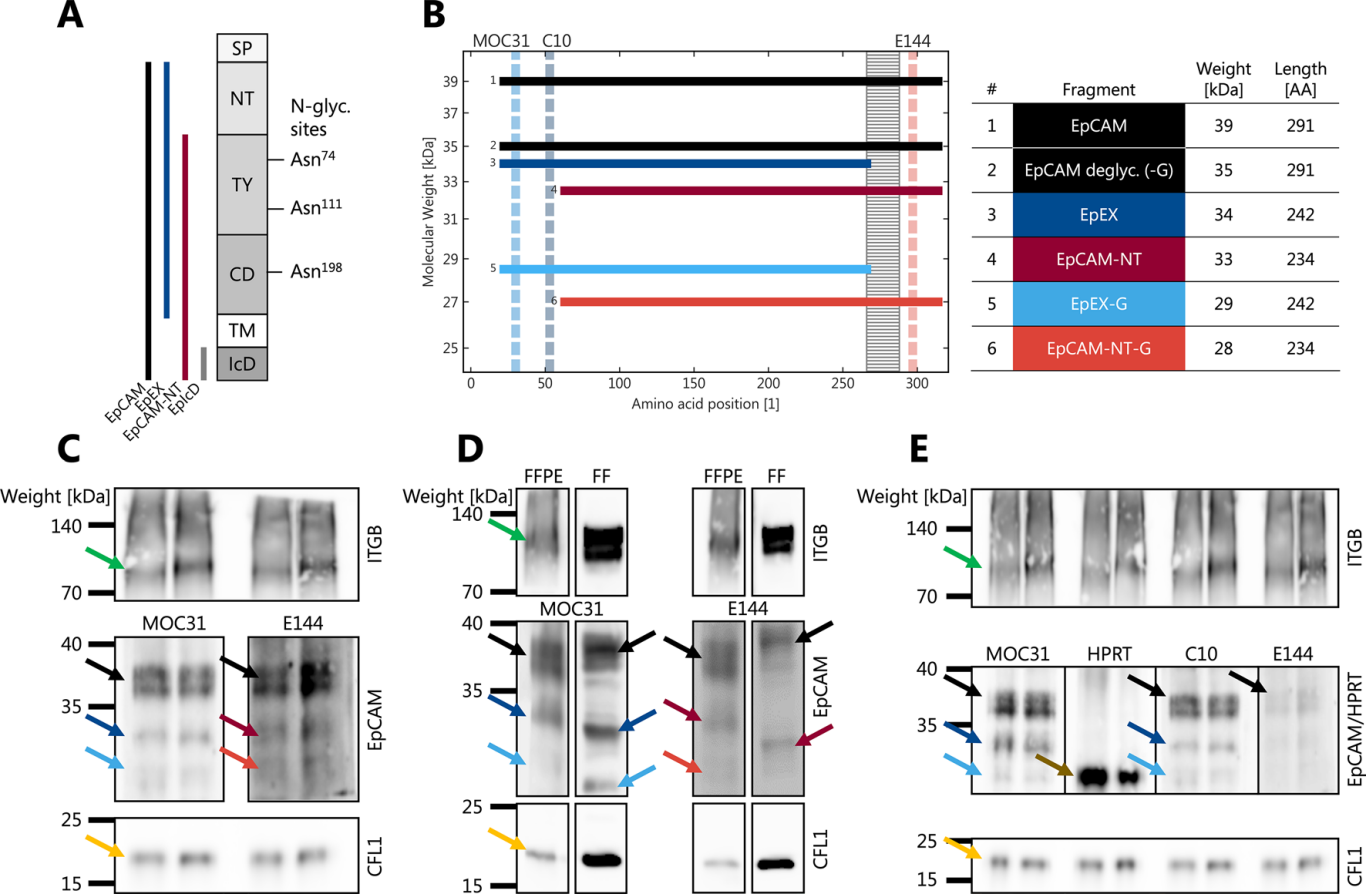
*Qualitative analysis of EpCAM fragments.* **A** EpCAM domains, fragments and glycosylation sites; *SP* signal peptide; *NT* N-terminal domain; *TY* thyreoglobulin-like domain; *CD* C-terminal domain; *TM* transmembrane domain; *IcD* intracellular domain; **B** Theoretical electrophoretic size distribution of the different variants and the binding sites of different antibodies (dashed vertical lines); hatched rectangle is TM domain; **C** Exemplary immunoblot from diagnostic formalin-fixed paraffin-embedded (FFPE) tissue with identification of the different variants using different primary antibodies as in subplot B; *ITGB* integrin beta, green arrow; CFL1 = cofilin-1, yellow; **D** Comparison of immunoblotted band patterns from FFPE and fresh-frozen cell lysates (FF); **E** Validation using an analogous antibody (C10) and a different target protein (HPRT; brown arrow) to estimate specificity

We evaluated the immunoblot fragment patterns of both diagnostic formalin-fixed paraffin-embedded (FFPE) tissue and unfixed protein extracts from fresh-frozen (FF) cells (Fig. 1). We observed only mild shifts in the apparent molecular weight between FF cells and FFPE tissue, with the exception of deglycosylated EpCAM. We observed a double peak at EpCAM 37 kDa with both MOC31 and E144, the lower of which we attributed to the deglycosylated variant—either only partially deglycosylated or with reduced electrophoretic migration compared to FF proteins. With MOC31, we observed two other bands at 34 kDa and 28 kDa, corresponding to EpEX and deglycosylated EpEX (EpEX-G). With E144 further bands were observed for EpCAM without its N-terminal domain (EpCAM-NT; 32 kDa) and its deglycosylated form (EpCAM-NT-G; 28 kDa). Due to its very small weight (2–3 kDa), EpIcD could not be identified.

To investigate the specificity of the immunoblotting approach, a non-EpCAM antibody against a protein of similar size was used (hypoxanthine–guanine phosphoribosyltransferase, HPRT; Fig. 1E) together with simple protein stains (not shown). Potentially confounding high-abundance protein bands within the respective size range were thus excluded. Validating the main primary antibody MOC31, a different clone (C10) with an analogous binding region demonstrated the same fragment size pattern (Fig. 1E). Of note, we observed a distinct additional band below 28 kDa (and above 25 kDa) with MOC31 in some samples.

Further antibodies were investigated but proved either unspecific or not sensitive enough in FFPE tissue. These are listed in the Methods section.

### Cohort characteristics

76 samples were quantifiable with at least one EpCAM variant and the loading control. The 52 tumor samples included 20 papillary non-invasive (pTa), 1 carcinoma in situ (pTis), 11 stroma-invasive (pT1), 5 muscle-invasive (pT2), and 15 advanced tumors (> pT2). Adjacent healthy bladder mucosa from the same case was available for 20 with another 4 samples of healthy mucosa without corresponding tumor.

### Qualitative patterns of EpCAM variants across the UC tumor spectrum

**Fig. 2:**
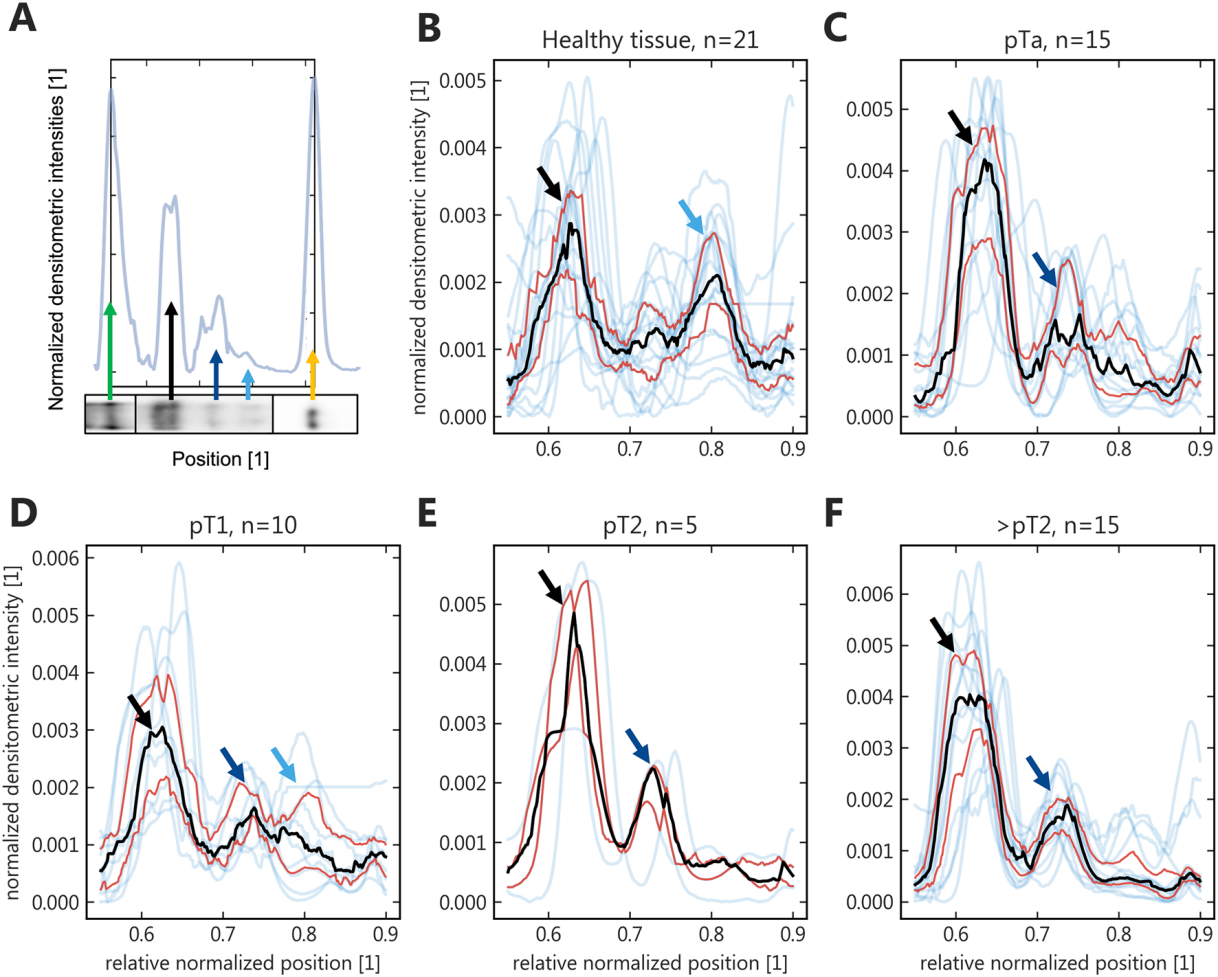
*Comparison of fragment size profiles across clinical urothelial cancer (UC) samples.* **A** Visualization of lane profile alignment using a high (ITGB; green arrow) and low-molecular-weight protein (CFL1; yellow) for normalization of the EpCAM bands/peaks in between (EpCAM = black; EpEX = dark blue; EpEX-G = light blue); **B**–**F** Profiles for UC samples of the respective histopathological stage (light blue); black = median; red = 25th and 75th percentile respectively

For the more sensitive band detection with MOC31, we compared the resulting band pattern by aligning the lane profiles by the upper (ITGB1) and lower (CFL1) end control peaks. Overlaying these profiles revealed stage-dependent changes (Fig. 2). Compared to healthy tissue, pTa tumors showed a prominent glycosylation of EpEX, with some retained EpEX-G in pT1 tumors, and a very homogenous pattern of EpCAM and fully glycosylated EpEX in muscle-invasive tumors. Also, relative dominance of EpCAM over EpEX was observed in all tumor stages compared to healthy tissue.

### Expression of EpCAM variants across the UC tumor spectrum

**Fig. 3:**
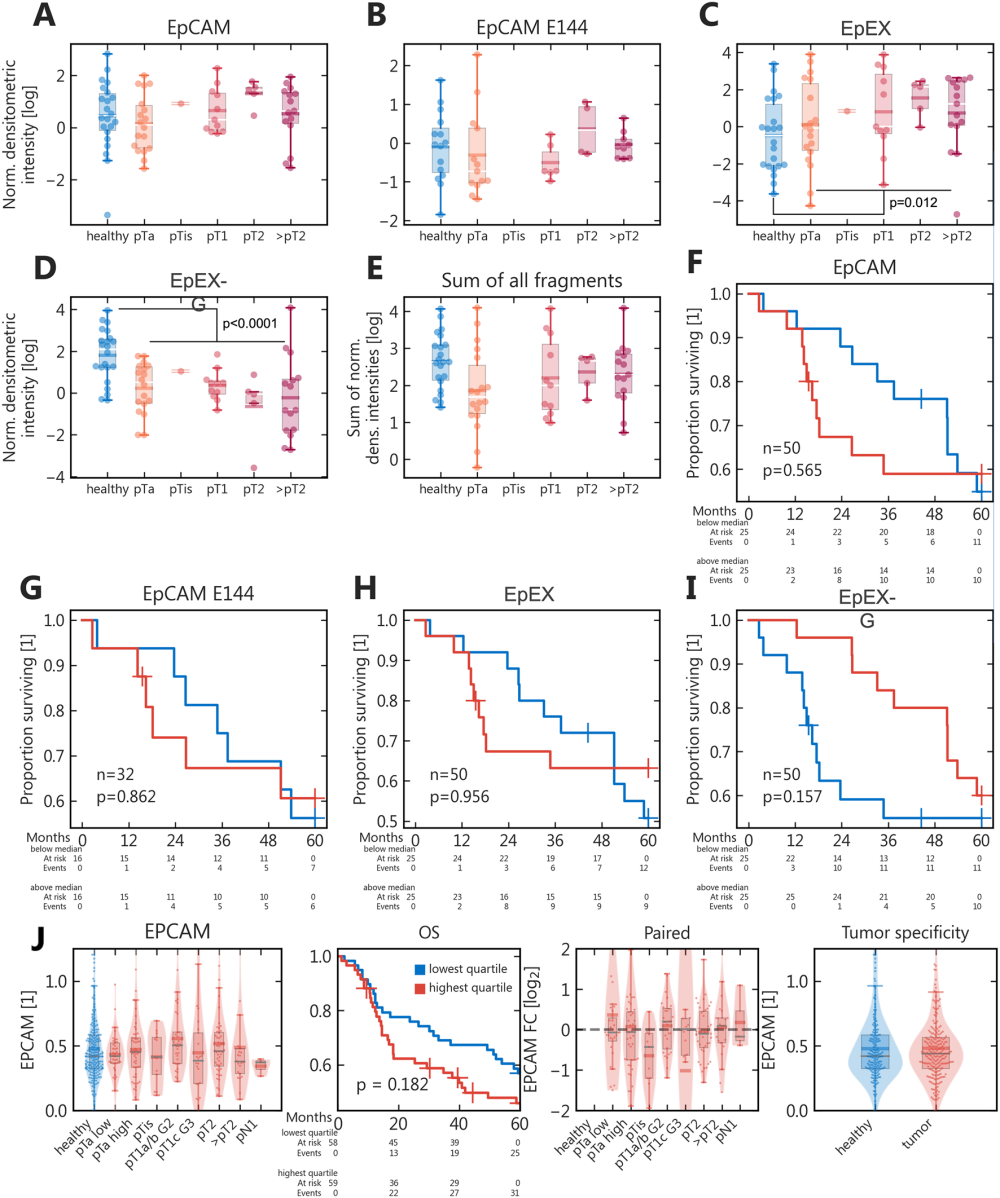
*Quantitative expression of EpCAM variants and survival analysis.* **A**–**E** Log-expression across the respective histopathological stages; whiskers = 95%-interval; box = inter-quartile range; white line = median; colored line = mean; **F**–**I** Kaplan–Meier plots of overall survival stratified by protein expression; red = expression above median; blue = below median; p value from Wilcoxon-weighted log-rank test; **J** In analogy for overall EpCAM measured by LC–MS/MS

With a sufficiently broad and linear dynamic signal range (Supplementary Fig. S1), we evaluated the expression of EpCAM fragments quantitatively (Fig. 3). In accordance with the peak profiles, EpEX-G was significantly lower in tumor compared to healthy samples (1.24 [0.44–2.59] vs. 8.11 [3.40–13.31]; *p* < 0.0001; *n* = 50, 22), vice versa for glycosylated EpEX (1.63 [0.60–11.61] vs. 0.66 [0.12–3.70]; *p* = 0.012; *n* = 50, 22) and with inverse correlation between these two EpEX variants (Spearman’s rank correlation coefficient *r* = − 0.65; *p* < 0.0001; *n* = 72).

For EpCAM and the sum of all EpCAM fragments, no relevant differences were observed. Importantly, there was no indication of relevant tumor specificity, neither for EpCAM-NT and EpCAM-NT-G (supplemental Fig. S2) which were quantifiable in fewer samples due to the lower sensitivity of E144.

### EpEX-G shows a trend of early survival stratification

Comparing overall survival for samples above and below median expression showed no relevant survival stratification for EpCAM, EpEX, EpCAM-NT, and EpCAM-NT-G. For EpEX-G, however, survival was significantly stratified at 48 months (*p* = 0.025) with subsequent intersection of the survival curves (*p* = 0.173 with complete follow-up).

### Immunohistochemistry cannot be used for quantification of EpIcD

IHC was used on four representative samples to compare the cellular distribution of extracellular (MOC31) and intracellular (4A7; IHC analogue of E144) EpCAM epitopes (supplemental Fig. S3). With both antibodies, we detected membrane bound EpCAM but no specific nuclear signal, suggesting that IHC does not detect EpIcD in FFPE tissues.

### LC–MS/MS proteomics confirm a lack of stage or tumor specificity

Validating our results, we analyzed an extended overlapping UC cohort comprising 419 samples, thereof 234 tumor and 185 healthy samples across all stages (low-grade pTa 38; high-grade pTa 54; pTis 13; pT1 56; pT2 48; > pT2 22; pN1 3). In 28 tandem mass-tagged samples sets, EpCAM was identified with mean 3.3 unique peptides, mostly from amino acids 100 to 250, thus allowing no calculation of variant-specific abundances. EpCAM was not tumor-specific, neither across tumor stages, nor in a pooled comparison or by individualized expression profiles (Fig. 3J; Supplementary Fig. S2). Overall survival was not relevantly stratified either (ibid.).

### Extracellular EpEX does not promote proliferation in UC cell lines T24 and HT1376

**Fig. 4:**
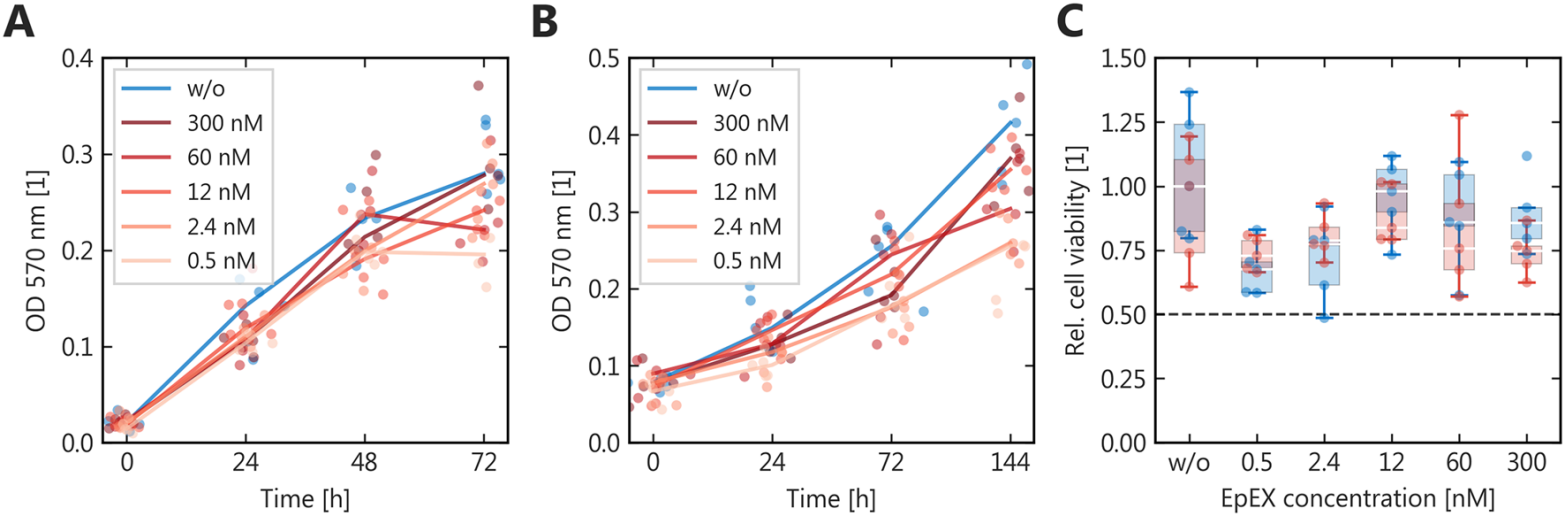
In vitro* proliferation effects of EpEX.* Proliferation measured by the MTT assay with different extracellular concentrations of EpEX for T24 (**A**) and HT1376 (**B**) urothelial cancer cells; (**C**): relative proliferation at the maximum incubation duration; red = T24; blue = HT1376; normed to the median control

Based on the marked reduction of EpEX-G levels in tumors and the increased expression of EpEX, we investigated whether addition of EpEX to cultured cancer cells would promote tumor growth. We measured cell proliferation and viability with the MTT assay using two UC cell lines from different stages (T24 and HT1376) (Zuiverloon et al. 2018). Neither cell line showed relevantly increased proliferation (Fig. 4), with a weak tendency of reduced proliferation in the low nanomolar range and likely unspecific inhibition ≥ 300 nM.

## Discussion

EpCAM has been used as target of both ADCC and ADCs in multiple cancers (Gottlinger et al. 1986; Schmidt et al. 2010, 2012; Eyvazi et al. 2018; Keller et al. 2019), with ongoing development in UC (Kowalski et al. 2010, 2012). While the mixed clinical effects could also result from suboptimal conjugated drugs or immune cell activation, efficacy primarily depends on the selective expression of the target antigen. We therefore systematically investigated and quantified the tumor specificity of EpCAM in UC—including its understudied proteoforms as possible confounders.

### Proteolytic EpCAM fragments can be identified in clinical UC samples

The different EpCAM fragments were originally identified in cell line models (Schnell et al. 2013a, b). We confirm the existence of these fragments in clinical FFPE samples. While EpCAM, EpEX, and EpCAM-NT matched well between FFPE and FF protein extracts, a reduction of the overall size range was observed in FFPE tissue. We attribute this to aberrant electrophoretic behavior of these cross-linked FFPE proteins and/or persistent partial glycosylation in clinical samples. The latter could be due to the importance of one of the three N-glycosylation sites for molecular stability (Munz et al. 2008). A fourth MOC31 band below 28 and above 25 kDa, which we observed in some samples, could be either non-specific or another, hitherto uncharacterized proteolytic fragment/glycoform.

### EpCAM is not tumor-specific in UC

Currently available data on EpCAM expression are based mostly on IHC (Momburg et al. 1987; Zorzos et al. 1993, 1995; Brunner et al. 2008; Schmelzer 2008; Fong et al. 2014). A few datasets include healthy normal mucosa. IHC is prone to degradation bias and lacks loading controls (Taylor and Levenson 2006). The former is particularly relevant in the urinary bladder as tumor necrosis and inflammation as well as different specimen types (cystectomy and transurethral resection) and sizes in diagnostic submissions are the rule, not the exception. Tissue and cohorts from different cases and patients are very likely to bias tumor specificity comparisons by differences in fixation and degradation. Immunoblot quantitation overcomes these limitations by providing loading controls that are similarly affected by preanalytical sample conditions. Additional mass spectrometric data provided method orthogonality and optimal dynamic range as well as higher quantitative accuracy.

Neither data type nor the pathological subtypes demonstrated general tumor specificity of EpCAM. In line with our data, EpCAM expression assessed by flow cytometry was highly heterogenous (Nini et al. 2020), which was similarly reported in a cross-cancer IHC analysis (Spizzo et al. 2011). Relevant survival stratification was not noted either for most fragments, in line with previous IHC data (Brunner et al. 2008). Prognostic significance of urinary EpCAM (Bryan et al. 2014) is most likely due to correlation with the amount of shredded tumor cells in higher grade tumors. Of note, EpEX-G showed a trend of early survival stratification with better survival in EpEX-G-high patients. This presumably mirrors the similarity to the EpCAM fragment pattern in healthy samples.

The previously reported clinical response with targeted cytotoxins in UC (Kowalski et al. 2010, 2012) has to be reviewed in light of the application method. Due to the direct instillation into the bladder, non-specific cytotoxicity might explain these effects in analogy to other non-targeted instillation therapies (e.g., BCG and mitomycin C). An application for drug approval in Europe has temporarily been withdrawn (EMA 2021) and was halted in the US (Sava 2022) by the manufacturer.

### EpEX glycosylation status could moderate its oncogenic potential

We observed an inverse relationship between EpEX and its deglycosylated variant EpEX-G, with the latter being considerably lower in tumors. This has similarly been indicated in FF tissue of head and neck squamous cell carcinoma (Pauli et al. 2003). The increased stability and half-life of glycosylated EpEX might be a tumor-favorable trait (Munz et al. 2008). EpEX has been shown to activate EGFR/ERK signaling (Brown et al. 2021) and to higher proliferation and migration rates of cancer cells (Lin et al. 2012; Liang et al. 2018). EpEX has also been linked to stabilizing PDL1 expression (Chen et al. 2020). Our in vitro evaluation, however, did not show increased proliferation upon stimulation with glycosylated EpEX. Whether specific therapeutic deglycosylation (currently not feasible) could exert a tumor-inhibiting effect remains unanswered.

## Conclusions

In urothelial cancer, EpCAM and its fragments should not be regarded as tumor-specific without prior patient-specific predictive testing. Glycosylation probably increases the oncogenic potential of EpEX in tumors and could contribute to the complex role of EpCAM.

## Supplementary Information

Below is the link to the electronic supplementary material.

Supplementary file1 (PDF 2077 KB)

## Data Availability

Access to the associated data will be granted on request to the corresponding author.
