# Supplementary material for: EpCAM tumor specificity and proteoform patterns in urothelial cancer
Source: J Cancer Res Clin Oncol. 2023 May 8;149(11):8913–22. doi: 10.1007/s00432-023-04809-9 (PMC10374485; doi:10.1007/s00432-023-04809-9)
Supplement: Supplementary file 1 — Supplementary file1 (PDF 2077 KB) [file 432_2023_4809_MOESM1_ESM.pdf]

Figure **S1**

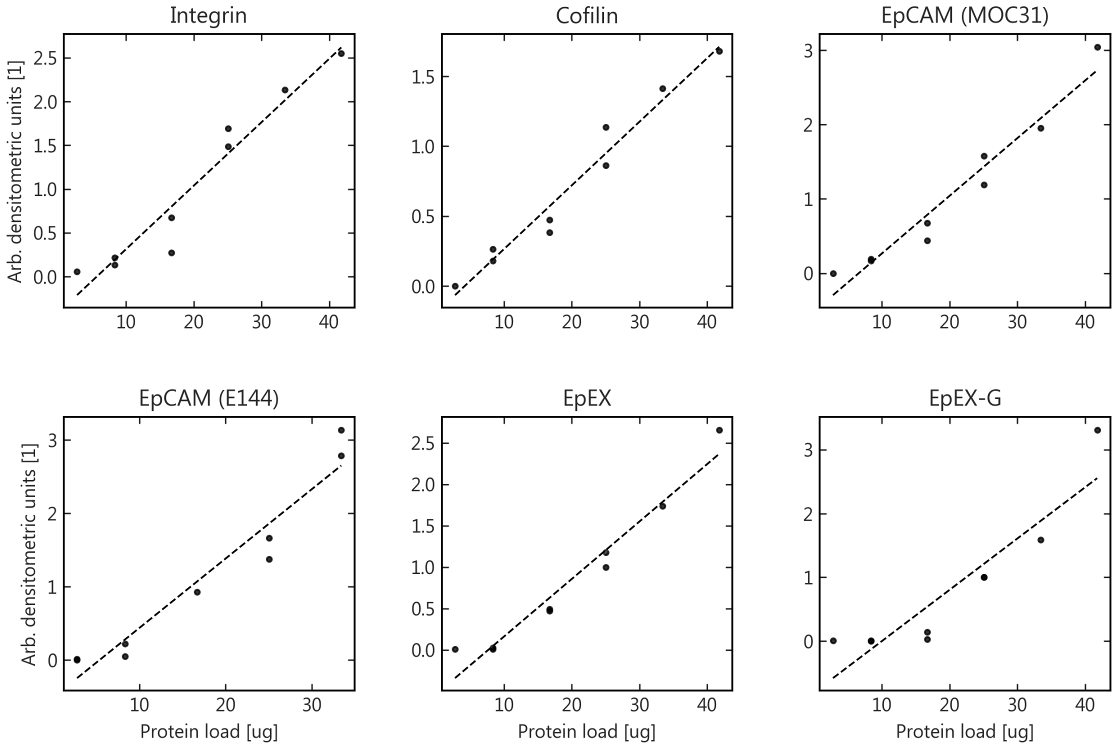

Supplemental Figure S1: *Dynamic range of the immunoblots.* Linear regression as dashed line.

Figure **S2**

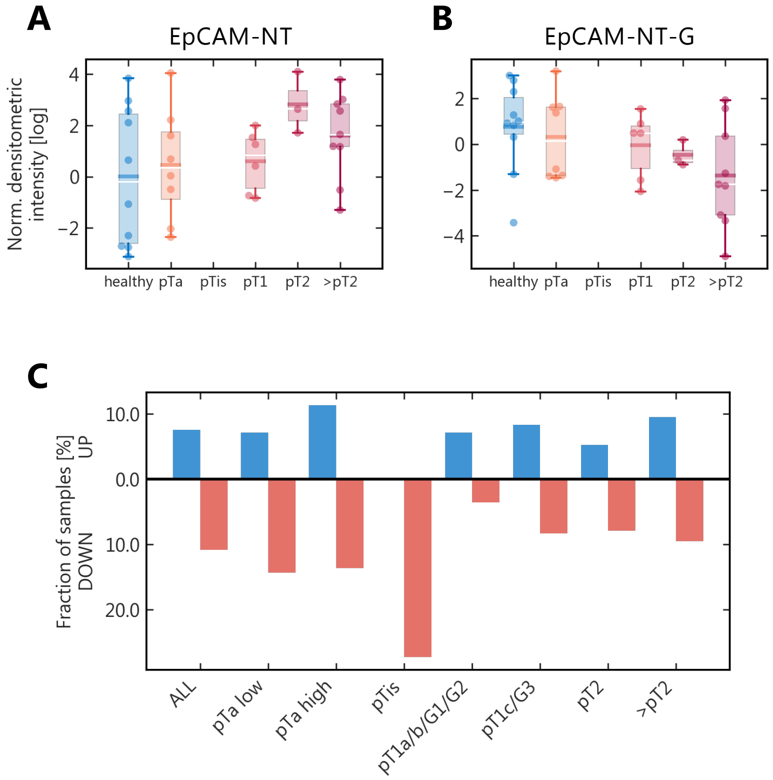

Supplemental Figure S2: *Additional data.* (A-B): Expression of the E144-quantified fragements EpCAM-NT and deglycosylated EpCAM-NT (EpCAM-NT-G); (C): Overall EpCAM up- and downregulation frequency across the LC-MS/MS validation cohort.

Figure **S3**

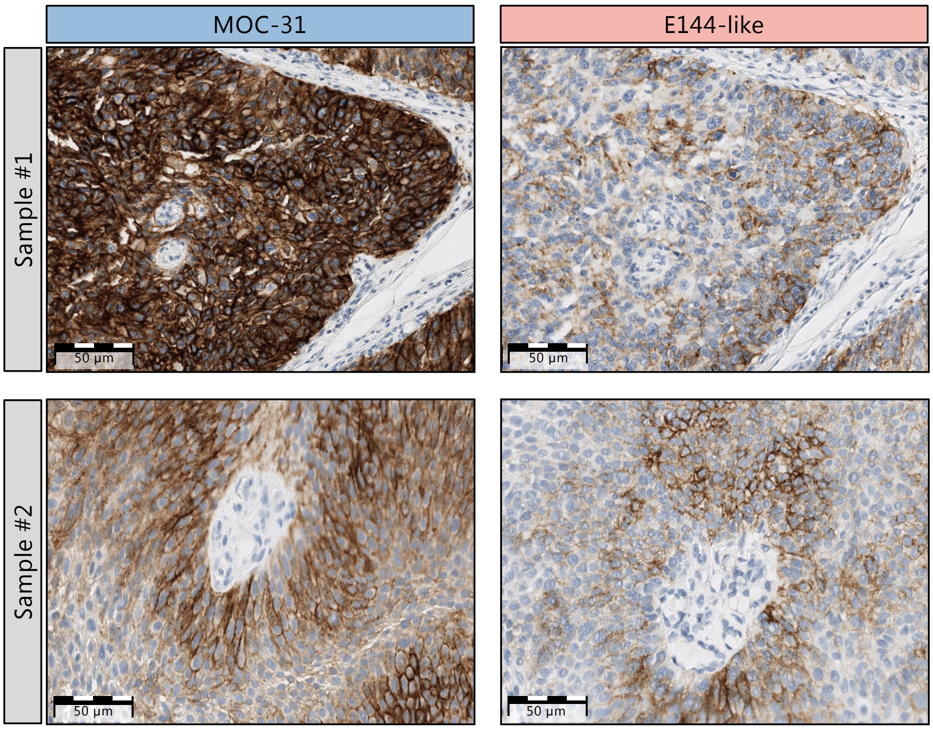

Supplemental Figure S3: *Immunohistochemistry (IHC) does not detect EpIcD*. Exemplary IHC images with membranous but no nuclear signal; digitalized with a 20-fold objective.
